# Diet

**Published:** 1835

**Authors:** 


### Art. IV.—Diet.

Doctor Bell, of New England, has lately written a spirited prize dissertation on diet, which has been published in the Boston Medical and Surgical Journal. It possesses some interest, although it embraces nothing new, being mostly a collection of the various facts already known to the profession. The doctor seems to be correctly opposed to the doctrine of some of the visionary lecturers of the "Land of steady habits," in his remarks on diet. After many reflections he arrives at the following conclusions: W. W.

1. A diet of *both* animal and vegetable food is adapted to the condition of the New England laborer.—2. No grand errors exist in his present system of diet, and no radical change is demanded to ensure a greater amount of health and strength, though many minor, but still important, errors exist.—3. The proportion of animal food usually customary is too great, and a considerable reduction would be expedient and advantageous, though it is impracticable to make a precise statement of the extent to which this is required, which must depend upon circumstances, as amount of labor performed, climate, season, bodily constitution, habits of life, &c. A general statement of this fact can alone be made.—4. The amount of food in general, customarily used, is more than is necessary for the maximum of health and strength, though a more specific statement of this abuse is also impossible. It must be left for each individual to attempt to reduce his quantity of food to that point at which he finds his mental and bodily powers most energetic. In searching for this point the New Englander may be almost certain that he must look for it in a descending ratio.—5. The great principle in regulating diet is to regard quantity rather than kind.—6. Perfect mastication and slow deglutition are important auxiliaries to the proper digestion of food.—7. A great variety of alimentary substances taken into the stomach at once, are calculated to do injury on several accounts.—8. The employment of alcoholic stimulants, and hot aqueous drinks, is deleterious to the functions of the stomach and to the general health.—9. The times of taking food, the state of the mental and moral functions, the quantity and times of exercise, &c. are all subjects of importance in the general subject of dietetics;—in these particulars, the habits of the New England laborer do not vary much from a healthful standard.

